# Using Real-Time Syndromic Surveillance to Analyze the Impact of a Cold Weather Event in New Mexico

**DOI:** 10.1155/2018/2185704

**Published:** 2018-02-14

**Authors:** Victoria F. Dirmyer

**Affiliations:** New Mexico Department of Health, Health Systems Epidemiology Program, Santa Fe, NM, USA

## Abstract

**Objective:**

This report describes the development of a novel syndromic cold weather syndrome for use in monitoring the impact of cold weather events on emergency department attendance.

**Methods:**

Syndromic messages from seven hospitals were analyzed for ED visits that occurred over a 12-day period. A cold weather syndrome was defined using terms in the self-reported chief complaint field as well as specific ICD-10-CM codes related to cold weather. A *κ* statistic was calculated to assess the overall agreement between the chief complaint field and diagnosis fields to further refine the cold weather syndrome definition.

**Results:**

Of the 3,873 ED visits that were reported, 487 were related to the cold weather event. Sixty-three percent were identified by a combination of diagnosis codes and chief complaints. Overall agreement between chief complaint and diagnosis codes was moderate (*κ* = 0.50; 95% confidence interval = 0.48–0.52).

**Conclusion:**

Due to the near real-time reporting of syndromic surveillance data, analysis results can be acted upon. Results from this analysis will be used in the state's emergency operations plan (EOP) for cold weather and winter storms. The EOP will provide guidance for mobilization of supplies/personnel, preparation of roadways and pedestrian walkways, and the coordination efforts of multiple state agencies.

## 1. Introduction

Extreme weather events, either hot or cold, can have both direct and indirect impacts on health. Increases in morbidity (falls due to snow and/or ice, carbon monoxide poisoning, and hypothermia) and mortality are observed during the winter months each year [1, 2]. In geographic regions where winter weather is not common, municipalities may be underprepared to deal with the associated health impacts of a major winter weather storm.

Countries, states, and municipalities have state emergency plans or emergency operation plans (EOP) that describe operations during emergency events like floods, tornados, fires, and winter weather storms. These documents outline how different groups, people, facilities, and so on, will respond during an emergency. Some jurisdictions have an EOP specific to winter related events like cold freezes or winter storms [3, 4], but many just rely on a generic EOP that applies to all emergencies. In New Mexico, the state has an all hazard emergency operations plan with functional annexes that apply to winter storms. The availability of syndromic surveillance data during emergencies would provide state officials with a view into what is happening at the local level. This is especially important for a large, rural state like New Mexico, where all government functions are centralized.

Syndromic surveillance systems were implemented in the early 2000s to increase the ability of state or local entities to detect events that would require immediate response, such as disease outbreaks or bioterrorist activities [5]. Federal legislation provided a mechanism for increasing the amount of data available for surveillance. One of the aims of the Health Information Technology for Economic and Clinical Health Act (HITECH Act) is to automate health records in the United States. Federal funding is provided to health facilities to facilitate the adoption of health information technology [6]. Now that syndromic surveillance has become a mainstay of many state and local health jurisdictions, new uses for syndromic surveillance data are being utilized. In some health jurisdictions, syndromic surveillance data is being used to characterize the current opioid crisis [7], monitor health conditions at mass gathering events [8], and understand the current burden of suicides [9] in different localities.

In New Mexico, 24 (67%) hospitals throughout the state provide syndromic surveillance data to the New Mexico Department of Health (NMDOH). In the southeast region of New Mexico, there are ten acute, nonfederal hospitals covering eight counties with an estimated population of 295,057. Data elements that are provided in syndromic messages include emergency department admission/registration information, self-reported chief complaint, discharge information, and diagnosis codes.

The southwest United States is commonly thought of as a region with warm temperatures and desert like conditions. In the southeast region of New Mexico, bordered by Mexico on the south and Texas on the east, average yearly snow fall is 10 inches. On December 26, 2015, a winter storm dropped 12+ inches of snow in this region over a 24-hour period. Wind gusts caused the formation of 12-foot snow drifts and white out conditions [10]. Drivers were not prepared for the force of the storm, and many became trapped in their vehicles on the side of the road. Many residents used their own equipment to clear roads as there was just not enough county and city officials or vehicles available to deal with the large amount of snow [11].

This report describes the development of a syndromic surveillance cold weather syndrome for use in describing the direct impact of a winter storm on emergency department visits. The objectives of this study are to assess the number of weather-related injuries associated with the cold weather event that occurred in southeast, New Mexico, using a combination of both the chief complaint field and diagnosis fields to create a cold weather syndrome and to assess the degree of agreement between these variables for refining the syndrome definition.

## 2. Methods

### 2.1. Syndromic Surveillance System

The Public Health Information Network (PHIN) guide [12], a national syndromic surveillance guide developed and published by the Centers for Disease Control and Prevention (CDC), provides guidance to facilities in the United States for submitting syndromic surveillance messages electronically to public health entities. The guide outlines a list of data elements that are required for syndromic surveillance. Emergency department (ED) visit data from hospitals participating in syndromic surveillance were selected for this study based upon two criteria: location in the southeast region of New Mexico and the inclusion of both the chief complaint field and diagnosis code fields. In the southeast region, there are 10 general, acute hospitals in this region. Seven of the ten facilities were providing syndromic surveillance data at the time of the cold weather event (72% coverage). Each emergency department visit can have up to 10 diagnosis codes, to include both diagnosis codes directly related to the illness or injury as well as external cause codes.

### 2.2. Cold Weather Syndrome

For this analysis, the following terms were used to identify ED visits related to the cold weather event from the chief complaint field: “fall,” “injury,” “sprain,” “knee,” “toe,” “wrist,” “arm,” “ice,” “snow,” “vehicle,” “MVA,” “MVC,” “laceration,” “frostbite,” “hypothermia,” and “suffocation” (indicative of CO poisoning). In addition to free text chief complaint terms, diagnosis codes* (International Classification of Diseases, Tenth Revision [ICD-10-CM])* [13] were also used to create the cold weather syndrome.  Table 1 outlines all ICD-10-CM codes that were used to identify ED visits related to the cold weather event. The codes selected have descriptions that follow a similar pattern to the phrases used in the chief complaint field. All terms and diagnosis codes were compiled to create one cold weather syndrome.  Table 1 includes diagnosis codes that apply to either the illness or injury of the patient and external cause codes (V–Y codes) that provide more information about the circumstances of the ED visit. For this analysis, an ED visit with a diagnosis code from Table 1 in any diagnosis code field (1–10) would flag the ED visit as related to the cold weather event.

### 2.3. Statistics

*κ* statistics [14] were used to assess the agreement between the chief complaint field and diagnosis code fields. Four comparisons were analyzed: incidents involving snow and ice, transport related incidents, falls related to snow and/or ice, and falls in general (the four groups were not mutually exclusive in the chief complaint field, but exclusive for diagnosis codes). All analyses were completed using Stata v.14.

## 3. Results

**Figure 1:**
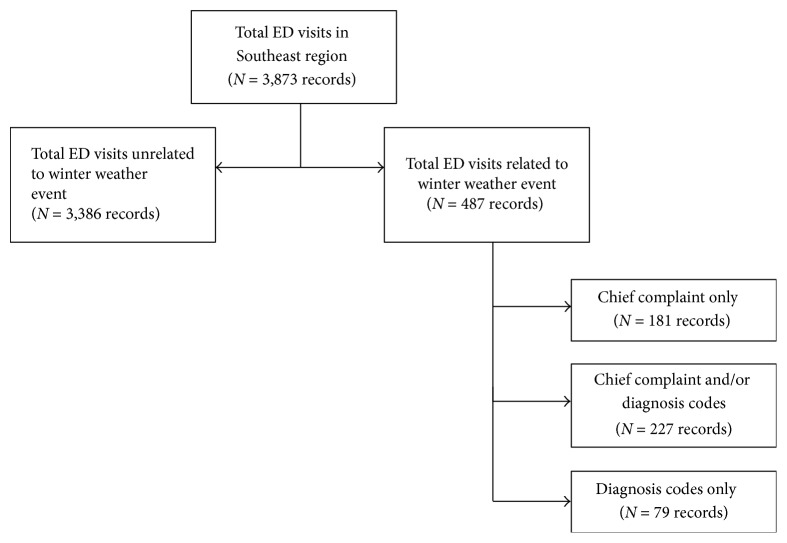
Number of cold weather-related emergency department visits detected through chief complaint and discharge diagnosis data in syndromic surveillance, New Mexico.

During the 12-day analysis period (December 26, 2015–January 6, 2016), 3,873 ED visits occurred in the southeast region of New Mexico. In total, 487 (12.6%) cold weather ED visits were identified by diagnosis code, chief complaint, or both (Figure 1). The majority of patients were male (*n* = 244, 51%) and aged 18–44 (*n* = 209, 43%). Of the 487 visits, 227 (47%) mentioned cold weather terms and/or codes in their chief complaint field and diagnosis fields, 181 (37%) mentioned cold weather terms in the chief complaint field and did not have diagnosis codes reported, and 79 (16%) visits had cold weather diagnosis codes but did not have cold weather terms in the chief complaint field. Twenty-nine percent of the ED visits had missing diagnosis information (Table 2).

For cold weather-related ED visits identified by diagnosis code, the majority of the ED visits (*n* = 113, 37%) had the code “W19” for unspecified fall (and associated sequelae codes). The second and third most observed codes were “W01” and “W00” for fall on same level from slipping, tripping, and stumbling (and associated codes) with 71 (23%) ED visits and falls due to ice and snow (and associated codes) with 56 (18%) ED visits, respectively. Overall there were 47 (17%) ED visits due to an injury involving transport of some kind (V00–V99).

For cold weather-related ED visits identified by chief complaint, regardless of the inclusion of diagnosis codes, there were 670 ED visits identified with cold weather terms. Of these 670 ED visits, 227 ED visits had both a chief complaint term and diagnosis code that identified the ED visit to be cold weather-related (Table 2). The most prevalent cold weather-related terms were “fall,” “injury,” and “MVA or MVC,” with 91 (40%), 72 (32%), and 30 (13%) ED visits, respectively. The majority of the 262 ED visits that only had a chief complaint with a cold weather term (no corresponding winter weather related diagnosis codes) had diagnosis codes for joint disorders, head injuries due to striking against or being struck by objects, or hand injuries caused by contact with a knife, sword, or dagger.

Of the 79 ED visits identified by cold weather-related diagnosis codes but no cold weather-related terms in the chief complaint, the most common terms were “CO_2_ exposure,” “CO_2_ poisoning,” “fell,” “back pain,” “ankle swelling,” “headache,” “head contusion,” “foot pain,” “swollen feet,” “ski accident,” “cold exposure,” “hit head,” and “shoulder pain.”

Overall agreement between chief complaint and diagnosis codes was moderate overall (*κ* = 0.50; 95% confidence interval [CI] = 0.48 to 0.52) (Table 3). The conditions with the highest agreement were transport related injuries (*κ* = 0.74; 95% confidence interval [CI] = 0.72 to 0.76) and lowest for injuries relating to snow and/or ice conditions (*κ* = 0.10; 95% confidence interval [CI] = 0.09 to 0.11).

## 4. Discussion

Timely detection of health events is critical for a syndromic surveillance system to monitor and detect public health issues. This study contributes to the limited literature on the use of ED syndromic surveillance data to monitor cold weather events. This study aimed to develop a cold weather syndrome that would assist in improving cold weather surveillance during the winter months and could supplement the state's cold weather emergency operations plan. The findings illustrated that the use of chief complaint alone would overestimate the number of cold weather-related ED visits because of the use of broad terms like “injury” that could occur any time and are not specific to cold weather events. The combined use of chief complaint terms and diagnosis codes provided for a better-defined cold weather syndrome.

For this cold weather syndrome analysis, the level of agreement between chief complaint and diagnosis codes was moderate at *κ* = 0.50. Concerning further analysis into stratification by 4 groups, snow and ice incidents, transport related incidents, falls involving snow and ice, and falls in general, the highest agreement was observed in traffic related incidents and the lowest agreement was observed in snow and ice incidents. The low agreement may be due to small numbers as 12 ED visits were identified by chief complaint as being related to snow and/or ice, but only 1 ED visit had a coordinating cold weather-related diagnosis code. Reviewing the data manually, this study relies heavily on diagnosis coding accuracy [15]. For patients who self-reported “CO_2_ poisoning” the associated diagnosis code was Z77.29, contact with and (suspected) exposure to other hazardous substances instead of a T58 code. A second example was a patient who self-reported falling on ice; the associated diagnosis code was W19 for an unspecified fall as opposed to W00 for fall due to snow and/or ice. For syndromic surveillance to be successful, analysts need to define syndromes both broadly and succinctly, which is not aided when diagnosis codes lack the proper specification. In October 2015, the switch from ICD-9-CM coding to ICD-10-CM coding occurred, providing an additional 55,000 codes. The adjustment from ICD-9-CM to ICD-10-CM codes may take some time before medical staff become familiar with all the specifics now available in the new coding system.

For this analysis, 12 days of data were included. Due to the timing of the storm (right after a major holiday), local resources available (snow removal equipment and personnel), and the ruralness of New Mexico, a decision was made to include additional days after the storm in the analysis in order to capture as many winter storm related injuries as possible. It took many days to remove snow from major roadways in southeast New Mexico [10]. Many rural roads (i.e., dirt) were not included for snow removal; residents cleared the roads using their own equipment. After the storm, freezing temperatures caused issues with ice on roadways.

The use of syndromic surveillance data to estimate the burden of weather-related events has proven beneficial [16–21]. In England, the Department of Health along with Public Health England, have created a cold weather plan, which aims to reduce morbidity and mortality due to severe cold weather through a series of cold weather alerts broadcasted to the area experiencing the hazardous weather and through public health actions such as long-term planning and preparation for these types of weather events and the mobilization of resources for emergency response [16]. As part of the cold weather plan, “cold weather indicators” were developed and integrated into routine surveillance in the country's Emergency Department Syndromic Surveillance System (EDESS). Similar actions can be planned for future cold weather events in New Mexico, where personnel and supplies can be mobilized ahead of time for such conditions and alerts to the public can be broadcasted.

In addition to cold weather events, syndromic surveillance data has been used to measure the impact of heatwaves. Elliot et al. [18] used syndromic surveillance data in conjunction with meteorological data to monitor peaks in both weather conditions and medical visits in both EDs and general practitioner's office visits. By historically analyzing the relationship between hot temperature days and when peaks in medical outlets occurred, the researchers could create a heatwave surveillance response plan that could be enacted for the next warm weather season. This plan included the mobilization of resources and the creation of heatwave-related messages to be publicly displayed on public health bulletins.

This analysis concentrated on injuries related to either falls or transport related injuries. Previous research has included respiratory conditions (asthma, trouble breathing) and cardiac conditions (myocardial infarction) in their analysis of weather-related conditions [16]. For this analysis, these conditions were not included as they had the potential to cause false positives in the relationship between chief complaint and diagnosis codes for cold weather-related incidents [16]. Future analysis could assess the overall agreement between these chief complaint terms and relation to weather-related conditions as defined in the diagnosis fields.

The results from this analysis will aid in the state's emergency operations plan for winter storms. Both planning within (a) EDs in terms of staffing and preemptive stocking of medications, equipment, and supplies needed to deal with cold weather-related injuries and emergency operations and (b) planning and preparation within the state's emergency operations center to coordinate efforts across many agencies. In times of major meteorological or environmental events, methods of communication may not be immediately available. The presence of an automated syndromic surveillance system can provide needed insight into what is happening at the local level. Community issued public health alerts can assist in individual decision making. In the case of the New Mexico winter storm, many citizens believed they would not be impacted by the cold weather. Many ended up being stranded on the side of the road due to low visibility and ultimately the formation of snow drifts. By issuing public health alerts, citizens will be better informed about weather conditions and ultimately make better informed decisions.

Moving forward, it would be beneficial for syndromic surveillance data to include triage notes and/or clinical impression as additional variables for analysis [22]. While self-reported chief complaint has proven to be beneficial for syndrome characterization, triage notes and clinical impression provide more context to the encounter. Currently, both triage notes and clinical impression are not required syndromic surveillance message elements per the PHIN guide, but other states have found them to be useful for syndrome characterization [22].

## 5. Limitations

Although there are many advantages to using (near) real-time ED visit data, there are several limitations related to the use of syndromic surveillance data. The quality and breadth of data suffer for the improved timeliness [23, 24]. Chief complaint is something that can be gathered at the time the patient registers with the ED staff. The addition of diagnosis codes to a patient's chart is added sometime later quite possibly long after the patient has been discharged. The time it takes to add diagnosis codes is facility specific with some facilities able to complete a patient record in 72 hours and other facilities may take up to 2 weeks [25]. Second, chief complaint is a free text field in syndromic surveillance messaging; therefore it is open to misspellings, user-specific abbreviations, or slang terms [26]. Third, only seven out of the possible ten hospitals in the southeast region of New Mexico submit syndromic surveillance messages to NMDOH; therefore the numbers presented in this analysis are most likely an underestimate of the number of ED visits due to cold weather events.

## 6. Conclusions

The present findings suggest that syndromic surveillance data can be a rich source of information. The results of this study are important for clinicians and emergency preparedness coordinators as they will aid in the refinement of an emergency operations plan for cold weather events, guiding emergency preparedness coordinators on the types of injuries that may occur and the types of conditions, living, traveling, and so on people may be involved in. Ultimately the incorporation of a cold weather syndrome in routine syndromic surveillance analysis will assist with targeted public health messages that will help New Mexico residents prepare in times of cold weather events.

## Figures and Tables

**Table 1:** List of ICD-10-CM codes for cold weather-related events.

ICD-10-CM Code	Description
T33	Superficial frostbite
T34	Frostbite with tissue necrosis
T58	Toxic effect of carbon monoxide (and associated codes)
T68	Hypothermia (and associated codes)
V00–V09	Pedestrian injured in transport accident
V10–V19	Pedal cycle rider injured in transport accident
V20–V29	Motorcycle rider injured in transport accident
V30–V39	Occupant of three-wheeled motor vehicle injured in transport accident
V40–V49	Car occupant injured in transport accident
V50–V59	Occupant of pick-up truck or van injured in transport accident
V60–V69	Occupant of heavy transport vehicle injured in transport accident
V70–V79	Bus occupant injured in transport accident
V80–V89	Other land transport accidents
V90–V94	Water transport accidents
V95–V97	Air and space transport accidents
V98-V99	Other and unspecified transport accidents
W00	Fall due to ice and snow (and associated codes)
W00.1	Fall from stairs and steps due to ice and snow (and associated codes)
W00.2	Other fall from one to level to another due to ice and snow (and associated codes)
W00.9	Unspecified fall due to ice and snow (and associated codes)
W01	Fall on same level from slipping, tripping, and stumbling (and associated codes)
W01.1	Fall on same level from slipping, tripping, and stumbling with subsequent striking against object (and associated codes)
W01.10	Fall on same level from slipping, tripping, and stumbling with subsequent striking against unspecified object (and associated codes)
W01.11	Fall on same level from slipping, tripping, and stumbling with subsequent striking against sharp object (and associated codes)
W01.19	Fall on same level from slipping, tripping, and stumbling with subsequent striking against other object (and associated codes)
W19	Unspecified fall (and associated codes)
X31	Exposure to excessive natural cold (and associated codes)
X37.2	Cataclysmic Storm: Blizzard (snow) (ice) (and associated codes)

**Table 2:** Comparison of chief complaint versus diagnosis codes for cold weather-related ED visits.

Diagnosis codes	Chief complaint	
	*Yes*	*No*
*Yes*	227	79
*No*	262	2,170
*Missing*	181	954

**Table 3:** Agreement between chief complaint and diagnosis codes related to cold weather events (*N* = 2,738 ED visits).

	Chief complaint and diagnosis codes	
	Kappa	95% CI
Snow and ice incidents	0.10	0.09, 0.11
Transport related incidents	0.74	0.72, 0.76
Falls involving snow and ice	0.17	0.16, 0.18
Falls in general	0.38	0.36, 0.40
Overall	0.50	0.48, 0.52
